# Sulcal pits of the superior temporal sulcus in schizophrenia patients with auditory verbal hallucinations

**DOI:** 10.3934/Neuroscience.2024002

**Published:** 2024-01-31

**Authors:** Baptiste Lerosier, Gregory Simon, Sylvain Takerkart, Guillaume Auzias, Sonia Dollfus

**Affiliations:** 1 Normandie Univ, UNICAEN, ISTS, EA 7466, 14000 Caen, France; 2 Aix Marseille Univ, CNRS, INT, Institut de Neurosciences de la Timone, Marseille, France; 3 CHU de Caen, Service de Psychiatrie, 14000 Caen, France; 4 Normandie Univ, UNICAEN, UFR santé, 14000 Caen, France; 5 Fédération Hospitalo-Universitaire (FHU-A^2^M^2^P), Normandie Univ, UNICAEN, UFR santé, 14000 Caen, France

**Keywords:** schizophrenia, auditory verbal hallucination, structural MRI, superior temporal sulcus, sulcal pits

## Abstract

Auditory verbal hallucinations (AVHs) are among the most common and disabling symptoms of schizophrenia. They involve the superior temporal sulcus (STS), which is associated with language processing; specific STS patterns may reflect vulnerability to auditory hallucinations in schizophrenia. STS sulcal pits are the deepest points of the folds in this region and were investigated here as an anatomical landmark of AVHs. This study included 53 patients diagnosed with schizophrenia and past or present AVHs, as well as 100 healthy control volunteers. All participants underwent a 3-T magnetic resonance imaging T1 brain scan, and sulcal pit differences were compared between the two groups. Compared with controls, patients with AVHs had a significantly different distributions for the number of sulcal pits in the left STS, indicating a less complex morphological pattern. The association of STS sulcal morphology with AVH suggests an early neurodevelopmental process in the pathophysiology of schizophrenia with AVHs.

## 1. Introduction

Schizophrenia is a chronic disorder with prodromal symptoms that emerge long before the onset of psychosis between the ages of 15–25 years and exhibits continuous evolution over time, thus supporting both neurodevelopmental and evolutionary processes [1]–[3]. Among the various symptoms, auditory verbal hallucinations (AVHs) occur in 70% of patients [4],[5] and may be linked to brain changes in regions involved in auditory and language processes (for example, see [6]). Concerning language, the left hemisphere lateralization highlights its central role in executing language functions [7], thus positioning it as the primary focus for studies on AVHs. The left superior temporal region and the left inferior frontal region have significant roles in AVHs [8]–[10]. Functional studies have suggested that the inferior frontal (IFS) sulcus and the superior temporal sulcus (STS) may be involved [8]–[10]. Several theories have explained AVHs, including Frith's, which stated that AVHs are due to a misattribution of inner speech involving the IFS and STS in particular [11]. Several groups have applied various techniques to study patients with AVHs, including voxel-based morphometry [12] and cortical thickness analysis [13]. These studies systematically report reductions in either gray matter or cortical thickness within the temporal and frontal areas, more specifically within the transverse temporal gyrus and superior temporal gyrus [14],[15], in the middle temporal gyrus [16],[17] or the inferior frontal gyrus [15],[17]. Moreover, the severity of AVHs was associated with significant reductions in gray matter volume in the left superior temporal gyrus, the left insula, and the left inferior frontal gyrus [12],[18],[19]. According to the Frith theory, these findings suggest that reduced gray matter volume in brain regions specialized in auditory and language processing may contribute to a deficit in accurate internal speech attribution [11]. In addition, abnormal functional activity has been observed in the frontotemporal areas of patients with AVHs [20]. Although the involvement of the superior temporal and inferior frontal regions in auditory hallucinations is well documented, the precise neurodevelopmental processes associated with these alterations are still unclear. Thus, a thorough evaluation of abnormalities of the superior temporal and inferior frontal sulci (IFS), particularly those likely to occur during early embryonic and fetal development, could provide crucial information on the neurodevelopmental origin of AVH.

Gyrification from the tenth week of gestation leads to the concurrent emergence of sulci on the surface of the brain [21],[22]. The individual sulcal patterns established at this point remain much like those observed in adulthood [23],[24]; therefore, the patterns that are detected in adults reflect the early in utero process and could lend support to a neurodevelopmental cause. Some studies in patients with schizophrenia have shown pattern modifications in different sulci [25]–[30], especially in the STS in participants with AVHs [30]–[32]. One group reported decreased gyrification in the bilateral STS in conjunction with resistant auditory hallucinations [31]; meanwhile, Plaze et al. described a right STS displacement that was dependent on the type of AVH (i.e., internal vs. external) [32]. These findings suggest that investigating sulcal pits in the temporal region, in conjunction with the frontal region, could elucidate the anatomical source of these hallucinations.

Sulcal pits are the deepest local regions of a sulcus and correspond to the first cortical folds [33],[34]. They represent the outcome of the first step of gyrification that begins around the tenth gestational week [21],[35]. Thus, these pits offer the advantage of being remnants of the earliest establishment of the first cortical folds. Focusing on these pits could sidestep later influences on the sulcus shape by potential environmental factors, further gyrification, or sulcal depth. The number of deep sulcal pits remains stable after birth [36],[37], potentially reflecting early developmental constraints on the brain [35]. Moreover, sulcal pits and cortical peaks that are formed earlier are subject to stronger genetic influences as compared to later folds [38]. The STS, observed as early as 23 weeks' gestation [21], is therefore an area which is likely to have a significant genetic influence. The inferior frontal sulcus becomes apparent later, around the 28th week of gestation. Because of this quite early establishment of sulcal pits, differences between people with and without schizophrenia and AVHs could imply that these conditions originate in the earliest stages of gyrification. To date, sulcal pits have not been investigated in patients with schizophrenia and AVHs.

In the present study, we compared STS and IFS morphology between patients with schizophrenia and current or past AVHs and unaffected volunteers. Our hypothesis was that patients with schizophrenia and AVHs would have a different sulcal pit organization, with fewer sulcal pits in the left STS and IFS as compared to healthy participants, thus suggesting the influence of a process in early neurodevelopment in crucial language areas.

## 2. Materials and methods

### 2.1. Participants

This study included 53 patients with a diagnosis of schizophrenia (per the Diagnostic and Statistical Manual of Mental Disorders-IV) that all experienced an AVH episode and 100 unaffected controls that were matched for sex, age, level of education, and handedness. Handedness was assessed with the Edinburgh inventory score [39], and those scoring ≥60 were considered right-handed. All patients were stabilized with no change in their treatment over the last month, and only one patient was unmedicated. The inclusion criterion for the control group was no history of psychotic disorders or substance dependence (including alcohol), which was assessed with a Mini International Neuropsychiatric Interview (MINI) [40]. Specifically, item L6a of the MINI addresses AVHs: “Have you ever heard things other people couldn't hear, such as voices?”, and the 53 included patients all endorsed this item, this indicating either current or former AVHs (Table 1). All participants were selected from the laboratory database. They were free of sensory deficits, including auditory deficits, other neurological disorders, and known brain abnormalities. Participants gave their informed, written consent, in accordance with the Declaration of Helsinki, and the local ethics committee (Comité de Protection des Personnes Nord-Ouest, France) approved each experimental protocol in which the subjects were included.

**Table 1.** Participant characteristics.

**Characteristics**	**Patients**		**Controls**		**χ²/F**	***p***
	n = 53		n = 100			
**% Male**	60.4	59.0	X²=0.027	0.869
**% right-handed**	83.0	88.0	X²=0.726	0.394

	Mean	SD	Mean	SD		

**Age, (y)**	37.2	9.1	34.8	9.7	F=2.120	0.147
**Illness Duration (y)**	11.7	7.4	N/A	-	-	-
**Chlorpromazine equivalent (mg/d)**	465.7	363.6	N/A	-	-	-

**Positive And Negative Syndrome Scale (PANSS)**

**P3 (Hallucinations)**	3.8	1.9	N/A	-	-	-
**Positive**	15.6	5.7	N/A	-	-	-
**Negative**	17.6	6.4	N/A	-	-	-
General	30.1	7.6	N/A	-	-	-
Total	63.2	15.9	N/A	-	-	-

### 2.2. Magnetic resonance image acquisition and image processing

Neuroimaging data was acquired using 3-T magnetic resonance imaging system (Intera Achieva 3T Quasar Dual camera, Philips Medical System) at Cyceron, which is a biomedical imaging center in Caen, France. We used the following morphological T1-weighted sequence: three-dimensional T1 turbo field echo, matrix size: 256 × 256 voxels with 180 contiguous sections, repetition time = 20 ms, echo time = 4.6 ms, inversion time = 800 ms, flip angle = 10°, field of view = 256 mm, and resolution = 1 mm3 isotropic.

### 2.3. Neuroimaging data analysis

Cortical reconstructions were generated from the imaging data using Freesurfer 6.0 (https://surfer.nmr.mgh.harvard.edu/fswiki/recon-all/). The technical details of these procedures have been previously described [41]–[43]. Morphometric data was produced using the FreeSurfer default processing stream (recon-all), which includes motion correction and averaging of T1-weighted images, removal of non-brain tissue, automated Talairach transformation, and segmentation of volumetric structures of subcortical white and deep gray matters.

### 2.4. Sulcal pit extraction

**Figure 1.**
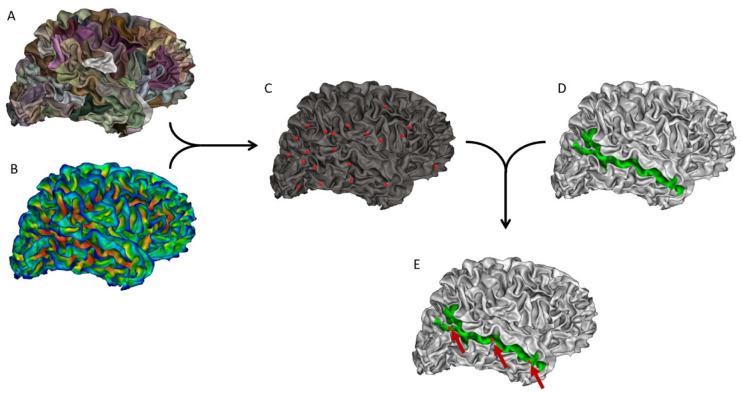
Example of all the steps for extracting the number of sulcal pits in the right superior temporal sulcus (STS). The same process was repeated in the left hemisphere, as well as in the left and right inferior frontal sulcus. A: Identification of several sulcal basins in each sulcus. B: Depth map to define depth values for sulcal pits. C: Identification of all the sulcal pits in the brain. D: Isolation of the STS according to Destrieux atlas. E: Identification of the sulcal pits in the STS only.

The sulcal pits correspond to the positions of local maximums in the depth of a sulcus. Here, the sulcal pits were extracted from the mesh of the white matter for each hemisphere, using a method implemented in BrainVisa 4.5 (https://brainvisa.info/web/). The whole process of sulcal pit extraction has been previously described in detail [33],[44].Briefly, the methodological pipeline involved the following steps for each person. A watershed algorithm divided large sulci into sulcal basins (Figure 1A). Simultaneously, a depth map was computed using the Depth Potential Function method introduced by Boucher et al. [45] (Figure 1B). Then, the deepest point of each sulcal basin was identified, and a watershed algorithm was applied to avoid the over- or under-extraction of sulcal pits (Figure 1C). We determined the depth of each sulcal pit as the value of the sulcal depth map at the pit location. The Destrieux parcellation atlas [46] was applied to the white matter mesh to isolate the STS region of interest (Figure 1D), which was used to delimit the zone of the pit extraction to solely the STS (Figure 1E).

**Figure 2.**
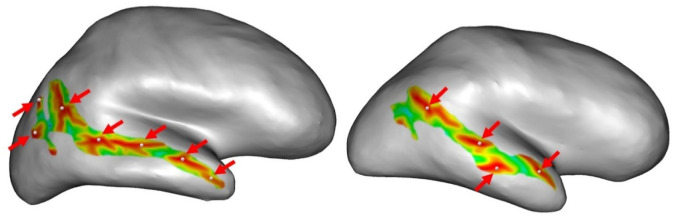
Example of inter-individual variability in sulcal pit detection in the right superior temporal sulcus. The right superior temporal sulcus is represented on the inflated surface by color. The red regions represent the deepest regions of the sulcus. The white sphere represents the detected sulcal pits. These inter-individual differences were investigated in the superior temporal sulcus and the inferior frontal sulcus of both hemispheres.

Finally, we obtained the number of sulcal pits for the left and right STS for each participant. The number of pits can differ from person to person, depending on the morphology of the STS (Figure 2).

### 2.5. Statistical analysis

Demographic differences for the proportions of males and right-handedness were either examined with Chi² tests or Mann–Whitney U tests for continuous variables. Because the data were not normally distributed, after confirming the absence of a relationship of sex or age with the number of sulcal pits, we used Fisher's exact tests to compare these counts for each hemisphere between the controls and the patients. Statistical analyses were performed using the RStudio software, version 3.5.2 (https://rstudio.com/).

## 3. Results

### 3.1. Participants

The patients group consisted of 53 persons, 32 men and 21 women (44 of them were right-handed and the mean age of the total population was 37.2 years (SD = 9.1)). The control group consisted of 100 persons, 59 men and 41 women (88 were right-handed and the mean age was 34.8 years (SD = 9.7)). There were no significant differences in age, sex, and handedness between the two groups. The average illness duration for the patients was 11.7 years (SD = 7.4), and the equivalent chlorpromazine dose was 465.7 mg per day (SD = 363.6). The average score of P3 hallucination items of the positive and negative syndrome scale (PANSS) for patients was 3.8 (SD = 1.9). For the positive, negative, general, and total PANSS, the average scores were 15.5 (SD = 5.7), 17.6 (SD = 6.4), 30.1 (SD = 7.6), and 63.2 (SD = 15.9), respectively.

### 3.2. Sulcal Pits in the STS

**Figure 3.**
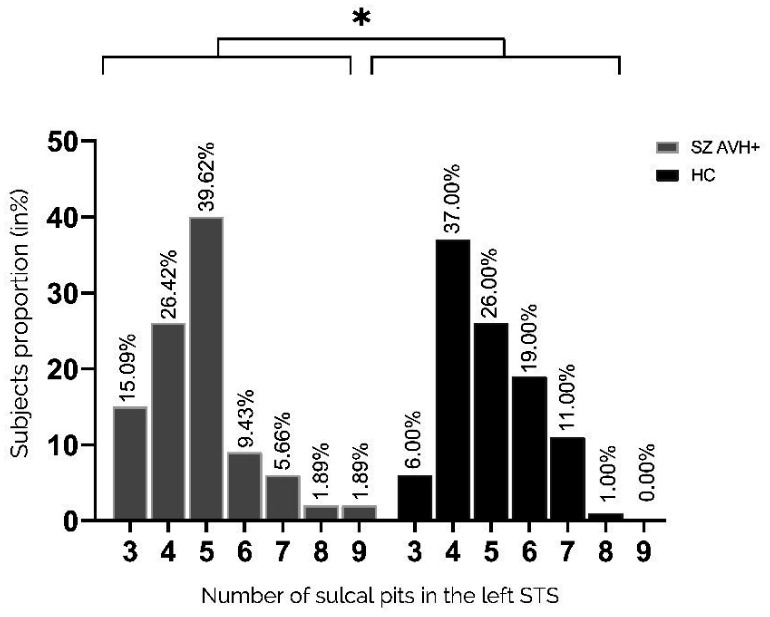
Distribution of pit numbers in the left STS. Proportions of participants with a given number of sulcal pits in the left STS (3–9 pits). In the left hemisphere, the distributions of pit numbers significantly differed between controls and patients (p = 0.05, Fisher's exact test).

**Figure 4.**
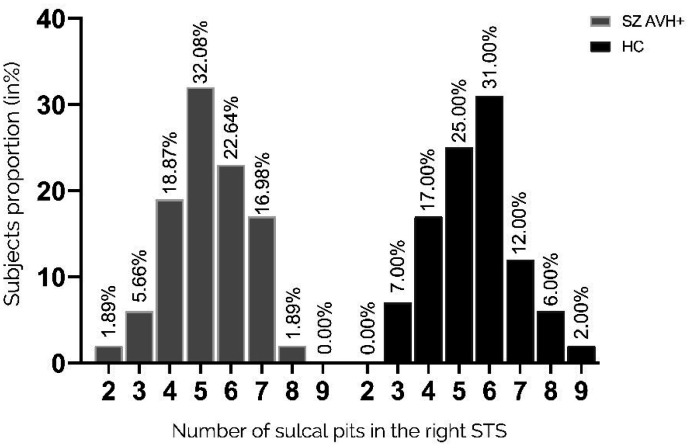
Distribution of pit numbers in the right STS. Proportions of participants with a given number of sulcal pits in the right STS (2–9 pits). Pit distribution in the right STS did not differ between the two groups.

The number of pits in the left STS significantly differed between the two groups (p = 0.05, Fisher's exact test) (Figure 3). Compared with patients, the unaffected controls had a higher proportion of participants with ≥5 pits (31% versus 18.9%), whereas the patient group had a greater proportion with exactly three pits (15.09% versus 6.0%). The pit distribution in the right STS did not differ between the two groups (Figure 4).

### 3.3. Sulcal Pits in the IFS

**Figure 5.**
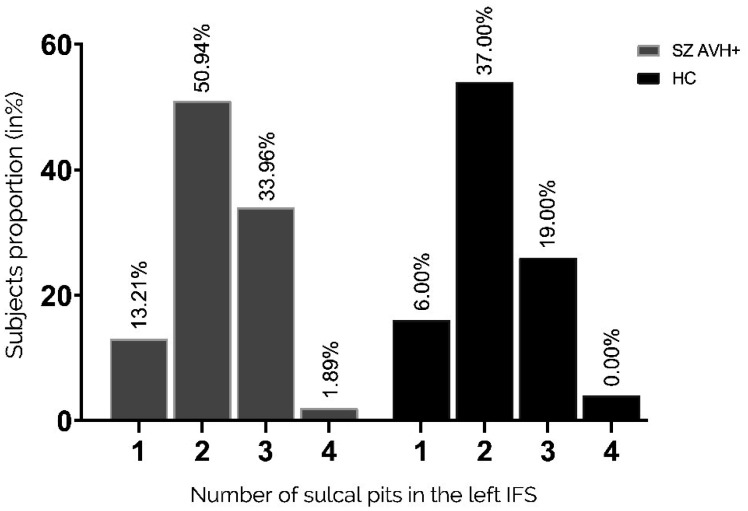
Distribution of pit numbers in the left IFS. Proportions of participants with a given number of sulcal pits in the right STS (1–4 pits). Pit distribution in the left IFS did not differ between the two groups.

**Figure 6.**
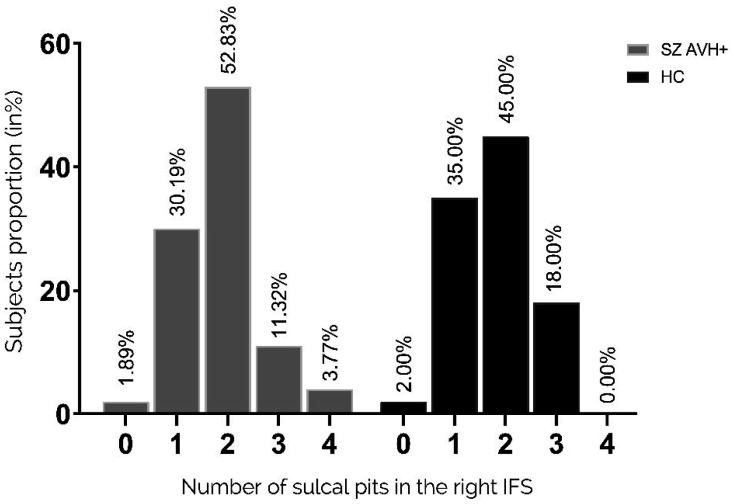
Distribution of pit numbers in the right IFS. Proportions of participants with a given number of sulcal pits in the right IFS (1–4 pits). Pit distribution in the right IFS did not differ between the two groups.

There was no significant difference in the distribution of pits in the left IFS between the two groups (p = 0.725 Fisher's exact test), as shown in Figure 5. Similarly, the distribution of pits in the right IFS showed no significant difference between the two groups, as shown in Figure 6 (p = 0.263 Fisher's exact test).

## 4. Discussion

Here, we describe the first findings of variations in sulcal pit patterns within the left STS in people with schizophrenia and a history of AVHs. The results suggest that the patients had an atypical morphological pattern with less complex gyrification in the left STS as compared with unaffected controls. A greater proportion of control participants had 5 or more sulcal pits in the left STS (median, 5; 19% vs. 31%, p = 0.05), whereas 2.5 as many patients had three pits when compared to the controls, which is the minimum number found within the sulcus. Pattern modification on the left side consistently supports that AVHs are related to language processing. These results are consistent with previous studies which showed that schizophrenia patients with AVHs presented a lower gyrification index compared to healthy controls [24],[47]. Under genetic influence, sulcal pits are considered the first landmarks established during cortical folding, [38],[48],[49]. Thus, the index we used here reflects very early brain morphological development and a likely biomarker of genetic vulnerability. Fewer sulcal pits implies fewer “pli de passage,” which is the transverse convolutions within a sulcus that connect the two bordering gyri. A greater number of these “pli de passages” indicates the presence of more axons linking functional areas on either side of the sulcus. Contrary to our hypothesis, there was no difference in the morphology of the IFS under genetic influence compared to healthy controls in both hemispheres. The later development of this sulcus could explain this absence of difference between the groups of participants. This suggests that a critical period around the 23 weeks of gestation could exist, which corresponds to the establishment of the STS. A study based on sulcal pit analyses demonstrated a specific cortical morphology in bipolar patients with a neurodevelopmental phenotype [50]. They exhibited a higher number of sulcal pits compared to bipolar patients without this neurodevelopmental aspect. Although that appears contradictory with our results, a modification of the sulcal pit patterns, whether an increase or a reduction, may come from a neurodevelopmental process. As mentioned, sulcal pits are indirect markers of brain connectivity. This means their quantity can be used as a proxy for interhemispheric connectivity, and a reduction in their number may indicate a potential reduction in interhemispheric transmission. Our findings align with recent functional studies that have demonstrated reduced interhemispheric communication due to white matter impairment within the interhemispheric language pathway in patients with schizophrenia and AVHs [51]. Additionally, different approaches can be used to investigate sulcal morphology organization such as sulcal pits depth. Brun et al. [52] established a correlation between the sulcal pit depth in the Broca's area and the severity of social communication difficulties among people with autism. Considering the involvement of a neurodevelopmental process in autism, these findings, alongside our own data, lead us to propose that a neurodevelopmental process may underlie schizophrenia with AVH.

If alterations in the STS prove to be reliable indicators of auditory hallucinations, the distinct configuration of the sulcal pits in the STS could serve as an early biomarker to identify vulnerabilities to auditory hallucinations in people at risk of schizophrenia. This could improve early diagnoses and enable more appropriate therapies to be provided more quickly. Moreover, it could lead to the application of specific treatments to reduce AVHs, such as repetitive Transcranial Magnetic Stimulation (rTMS).

This study had some limitations. First, we did not investigate all the sulci, but rather the STS which is the most involved in AVHs. Second, we did not compare patients with and without a history of AVH. In consideration of the established role of the STS in language processes, coupled with the absence of auditory hallucinations in individuals classified as such, we suggest that there may be potential differences in the number of sulcal pits between AVH- and AVH+ subjects, while expecting similarities between AVH- and control subjects. Otherwise, we cannot conclude that the particular sulcal morphology we identified is specific to AVHs. This might be the focus of future exploration; however, recruiting sufficient patients without AVHs is challenging, since their prevalence is much lower (30%) than that of patients with AVHs (70%). Third, the patients were in a relatively advanced stage of their illness. A longitudinal study in patients experiencing their first psychotic episode with AVHs might investigate the stability of this potential biomarker.

In conclusion, the present study provides the first demonstration of a specific pattern of sulcal pits in the left STS of patients with schizophrenia experiencing AVHs. These early-developing morphological characteristics in a region involved in language processing could represent the origin of vulnerability to AVH in schizophrenia. Further studies are needed to examine the potential of STS sulcal pits as biomarkers and their relevance to the pathophysiology of AVHs.
